# Advances in screening hyperthermic nanomedicines in 3D tumor models

**DOI:** 10.1039/d3nh00305a

**Published:** 2024-01-05

**Authors:** Joana F. Soeiro, Filipa L. Sousa, Maria V. Monteiro, Vítor M. Gaspar, Nuno J. O. Silva, João F. Mano

**Affiliations:** a Department of Chemistry, CICECO – Aveiro Institute of Materials, University of Aveiro, Campus Universitário de Santiago 3810-193 Aveiro Portugal vm.gaspar@ua.pt nunojoao@ua.pt jmano@ua.pt +351 234370733; b Department of Physics, University of Aveiro, Campus Universitário de Santiago 3810-193 Aveiro Portugal

## Abstract

Hyperthermic nanomedicines are particularly relevant for tackling human cancer, providing a valuable alternative to conventional therapeutics. The early-stage preclinical performance evaluation of such anti-cancer treatments is conventionally performed in flat 2D cell cultures that do not mimic the volumetric heat transfer occurring in human tumors. Recently, improvements in bioengineered 3D *in vitro* models have unlocked the opportunity to recapitulate major tumor microenvironment hallmarks and generate highly informative readouts that can contribute to accelerating the discovery and validation of efficient hyperthermic treatments. Leveraging on this, herein we aim to showcase the potential of engineered physiomimetic 3D tumor models for evaluating the preclinical efficacy of hyperthermic nanomedicines, featuring the main advantages and design considerations under diverse testing scenarios. The most recent applications of 3D tumor models for screening photo- and/or magnetic nanomedicines will be discussed, either as standalone systems or in combinatorial approaches with other anti-cancer therapeutics. We envision that breakthroughs toward developing multi-functional 3D platforms for hyperthermia onset and follow-up will contribute to a more expedited discovery of top-performing hyperthermic therapies in a preclinical setting before their *in vivo* screening.

## Abstract 2

Integrating the advances of emerging hyperthermia techniques with 3D tumor models and non-invasive temperature control systems can contribute to identifying top-performing hyperthermic nanomedicines in preclinical evaluation stages.

## Graphical abstract

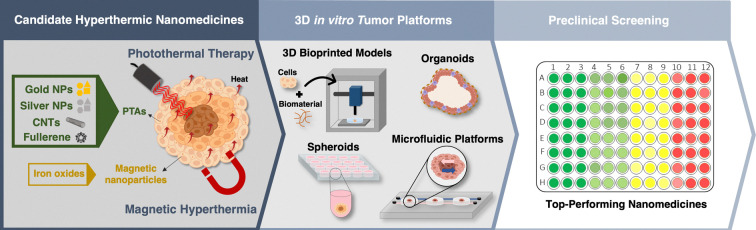

## 1 Introduction

Hyperthermia for cancer therapy is continuously evolving as a valuable strategy compared to standard chemotherapeutic treatments owing to its potential for heating tissues to induce cancer cell death and increase the immune response in a more controlled and localized mode.^1,2^ As cancer cells are more susceptible to heat damage than normal cells, hyperthermia can induce cancer cell death with minimal injury to the healthy tissue surrounding the tumor.^3,4^ The damage caused in cells depends on the achieved temperature and the duration of the procedure.^5^ Three ranges can be defined: 40–42 °C (mild hyperthermia), 42–45 °C (moderate hyperthermia), and ≥50 °C (ablation).^6^ Thermal ablation is usually performed for short periods and causes irreversible cell damage by inducing apoptosis.^6,7^ Mild and moderate hyperthermia are performed for longer periods, inducing changes in the blood perfusion and oxygenation of the tissue, causing protein denaturation and aggregation. Furthermore, mechanisms of DNA repair and cell proliferation can be inhibited, ultimately altering the physiology of the tumor.^7,8^ Recent evidence pointing toward hyperthermic activation of immune cells and increased resistance against secondary tumors further supports the validity of such hyperthermic strategies.^9^

In recent years, different types of nanomaterials approved by the Food and Drug Administration (FDA) for hyperthermia have been explored in the clinical setting (*e.g.*, Aurolase®, Nanospectra Biosciences, Inc., Houston, TX; and NanoTherm®, MagForce AG, Berlin, Germany) since they can be engineered to accumulate in the desired area and generate heat upon an external stimulus, typically an electromagnetic wave or an alternating magnetic field (AMF).^10^ Thus, nanomaterials can improve selectivity to prevent major injury in the tissues surrounding the tumor.^11^ Despite relevant developments, hyperthermia mediated by nanomaterials still faces several challenges, such as guaranteeing maximum cancer cell selectivity, homogeneous heating of the target tissue, and effective methods for temperature control/real-time readout during treatments.^12^ Consequently, current hyperthermic strategies require further engineering and improvements of 3D tumor models prior to their widespread acceptance as a standard-of-care therapeutic modality.^13^

Conventionally, the preclinical validation of candidate hyperthermic nanomedicines has been routinely performed in gold-standard flat 2D cell cultures.^14,15^ However, these are unable to recapitulate the 3D architecture of the human tumor microenvironment (TME), as well as the complexity of its cellular (*i.e.*, cancer, stromal, immune cells, *etc.*) and non-cellular elements (*i.e.*, extracellular matrix (ECM)),^16^ ultimately leading to a sub-optimal *in vitro*/*in vivo* correlation that impacts human clinical trial validation stages and ultimately translation into the market.^17,18^ Exploring 3D *in vitro* tumor models as alternative preclinical platforms for hyperthermic nanomedicines validation opens the possibility to recapitulate: (i) human solid tumors gene expression profiles associated with heat/drug resistance (*i.e.*, heat-shock proteins – Hsp70/Hsp90,^14,19^ drug resistance mechanisms – ABC transporters^20^), (ii) the modulation of the secretion of key exosomes,^21^ as well as (iii) the establishment of hypoxic/necrotic regions and volumetric pH gradients,^22^ in an approach that is significantly more similar to that found *in vivo*.^23^ The coexistence of multiple cancer-stroma cellular populations and the dynamic cell–cell or cell–TME interactions in 3D can also be explored to better contribute to evaluating hyperthermic therapeutics performance.^24–28^ Upgrading from the conventional use of monolayers toward volumetric tumor models also enables researchers to specifically evaluate the influence of solid tumors on heat transfer mechanisms and nanomedicines penetration in 3D in a more biomimetic set-up.^18,26,29^ The latter is particularly relevant, since a sub-optimal and non-homogeneous distribution of hyperthermic nanomedicines within the tumor volume may impact the overall therapeutic outcome. Leveraging 3D models and high-throughput/high-content imaging approaches to improve the selection process of top-performing hyperthermic nanomedicines at preclinical stages also contributes to reducing the use of laboratory animals and surpasses major ethical and economic issues associated with these models.^24^

Despite the promising advantages of advanced 3D tumor models, these have yet to be broadly adopted during hyperthermic nanomedicine design and performance screening.^30^ Aiming to shed light on recent advances, the key aspects of hyperthermia nanomedicines validation in advanced 3D *in vitro* models are herein addressed. An informative discussion focusing on the importance of heat transfer simulations in 3D and real-time temperature evaluation strategies during treatment is also provided, considering the highly required advances in technologies to monitor hyperthermia nanomedicines in a non-invasive mode. State-of-the-art examples leveraging on the use of 3D *in vitro* tumor models for screening and validating nano-hyperthermia technologies will be showcased and discussed, considering their contribution to further consolidate and upgrade this therapeutic methodology. It is envisaged that advances from upgraded preclinical validation models will contribute to changing the current approaches for the validation of innovative hyperthermic nanomedicines, opening new avenues for accelerating their translation toward the clinical scenario.

## 2 Hyperthermia technologies

**Fig. 1:**
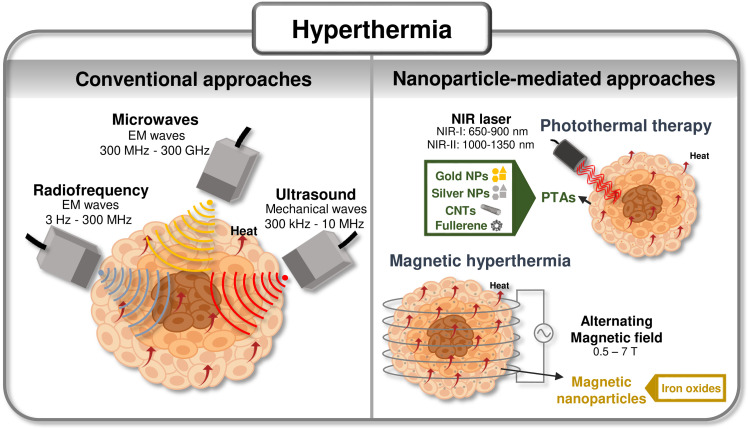
Summary of hyperthermic technologies: Scheme of currently available hyperthermic technologies and advanced approaches leveraging nanomaterials as heating agents. Compiled from ref. 11, 31 and 36–38.

Conventional hyperthermia for cancer management is performed by exploiting: (i) electromagnetic waves: radiofrequency (RF), microwaves (MW), near-infrared (NIR) light, and (ii) mechanical waves, namely ultrasound (US).^31,32^ Even though these methods are efficient in increasing the temperature of tissues, they fail to target only the desired tissue.^11^ This targeting can be dramatically enhanced using nanosized heating agents that are more efficient than tissues in absorbing the incoming wave, leading to an increase in temperature in a specific region. Hyperthermia has been rapidly emerging owing to its higher precision and therapeutic versatility, particularly in the case of photothermal therapy (PTT) and magnetic hyperthermia (MH), complementing the available toolset of hyperthermia technologies.^33–35^Fig. 1 highlights examples of traditional hyperthermia methods and examples of nanomaterials used as heating agents in hyperthermia procedures.^11,31,36–38^

### 2.1 Photothermal nanotherapy

In PTT approaches, nanosized photothermal agents (nano-PTAs) are leveraged to convert electromagnetic radiation, usually NIR, into thermal energy to heat cancer cells.^39^ The interest in NIR-II-absorbing materials (NIR-II: 1000–1350 nm^40^) has recently increased since they show a reduced light scattering, enable a higher spatial resolution, and higher signal-to-background ratio compared to NIR-I-absorbing materials (NIR-I: 650–900 nm^40^) which have been the most explored for PTT.^41,42^

To date, a wide range of inorganic (*i.e.*, carbon based-nanomaterials, noble metal nanoparticles (NPs), metal-oxide NPs), and organic nanomaterials (*i.e.*, conjugated polymers, semiconducting polymers, organic dyes, *etc*.) have been exploited for PTT methodologies.^11,43,44^Table 1 reports examples of nano-PTAs recently used for hyperthermic nanotherapy screening in 2D *in vitro* models. Both classes of nano-PTAs present advantages and disadvantages, with most inorganic nano-PTAs being non-biodegradable and exhibiting limited biocompatibility, while organic nano-PTAs (*i.e.*, biodegradable, and biocompatible) commonly exhibit lower photothermal conversion efficiency (PCE) and reduced photostability.^45–47^ The rationale selection of nano-PTA should thus account for several factors, including the type of cancer, the tissue/organ being treated, the location of the treatment, and the optimal light wavelength.

**Table 1:** Examples reporting the performance of nano-PTAs for 2D *in vitro* PTT

Heating agent			PCE^b^ (%)	Heating conditions	2D tumor model	Dose (μg mL^−1^)	*T* reached (°C)	Cell death (%) (time after treatment, technique)	Ref.
Core	Coating	Size w/coating (nm)							
SCNTs and MCNTs loaded with therapeutic siRNA	Peptide lipid and sucrose laurate	MCNTs: length: 0.5–2 μm; diameter: 30 nm; SCNTs: length, 1–3 μm, diameter: 24 nm, TEM	SCNTs ∼ 59	808 nm, 1 W cm^−2^, 5 min	HeLa	30	42–45	SCNTs: ∼30^a^	48
			MCNTs ∼ 58					MCNTs: ∼40^a^ (PTT alone) (24 h, MTT)	
MCNTs	Amide	Length: 591	N.D.	1064 nm, 3 W, 5–45 s	HMLER^shEcadherin^, HMLER^shControl^	50	49	∼40^a^ (for both cell lines) (24 h, MTT)	49
		Diameter: 29							
		Technique: N.D.							
DPP NPs	TPA	∼76, DLS	∼35 (80 μg mL^−1^)	660 nm, 0.5 and 1 W cm^−2^, 5 min	HeLa	20	N.D.	0.5 W cm^−2^: ∼30^a^; 1 W cm^−2^: ∼70^a^ (48 h, MTT)	50
Black mesoporous silicon NPs	PEG	156, DLS (w/o coating)	∼33 (100 μg mL^−1^)	808 nm, 1 W cm^−2^, 10 min	CT26	75	N.D.	84 (12 h, FC)	51
Chitosan-based hydrogels with nanosized {Mo_154_}	—	3.4^c^ {Mo_154_}, technique: N.D.	N.D.	808 nm, 0.8 W cm^−2^, 10 min	M21	0.092 wt%	N.D.	∼98 (after irradiation, CellTiter Glo)	52
Gellan Gum and Ca^2+^ hydrogels with AuNRs	PEG	N.D.	∼21 (100 μg mL^−1^)	(1) 808 nm, 0.5 W cm^−2^, 2 min; and (2) 660 nm, 50 mW cm^−2^, 5 min	HeLa and MCF-7	50	66.2	∼44 and ∼66 for HeLa and MCF-7 cells, respectively (12 h, CCK8)	53
Ag NPs	PVP	∼93, DLS	N.D.	970 nm, 3 W, 70 s	MDA-MB-231	12.5	52.4	>85 (72 h, MTT)	54
Au25 nanoclusters conjugated with ICG	Gluta-thione	∼3.4, DLS	N.D.	808 nm	MCF-7	30 μM	N.D.	0.5 W cm^−2^: ∼35^a^	55
				0–1 W cm^−2^				0.8 and 1 W cm^−2^: ∼100^a^ (after irradiation, MTT)	
				5 min					

a Value extracted from graphs.

b The conditions that are not indicated are the same as the heating conditions; N.D. – non described; PCE: photothermal conversion efficiency; MCNTs: multiwalled carbon nanotubes; SCNTs: single walled carbon nanotubes; DPP: diketopyrrolopyrrole; TPA: triphenylamine; PEG: polyethylene glycol; FC: flow cytometry; NRs: nanorods; PVP: polyvinylpyrrolidone.

c Value from the literature indicated in the article.

Carbon-based materials, including graphene,^56–58^ carbon nanotubes (CNTs),^59,60^ fullerenes,^61,62^ and carbon dots,^63,64^ have been increasingly used as nano-PTAs as they display high absorption in the NIR region, suitable biocompatibility, and can generally accumulate in the tumor site due to their nano size. Furthermore, these can be easily functionalized to improve their water dispersibility, biocompatibility, and tumor-targeting ability.^36,65^ Carbon-based nano-PTAs have the potential for multimodal imaging, integration of PTT with other therapies, such as photodynamic therapy (PDT), and can also be used as drug delivery vehicles.^66–68^

Noble metal NPs, such as gold (Au),^69,70^ silver (Ag),^54,71^ or palladium (Pd) NPs,^72,73^ have been shown to have an adequate performance as nano-PTAs due to their biocompatibility, suitable sizes for biological applications, and strong localized surface plasmon resonance that gives them the ability to be good absorption agents.^74,75^ These materials can be easily functionalized and conjugated with molecules to increase their targeting ability and biocompatibility.^76^ Furthermore, noble metal NPs can be used for imaging applications and combined therapies, such as PDT, immunotherapy, or chemotherapy.^77^ By tuning the size or shape of the structure, the absorption range can be easily changed.^75^

Organic dyes have strong light absorption in the visible or NIR region and can be easily synthesized and functionalized for specific applications.^50,78^ Furthermore, they can be simultaneously used as fluorescent probes for imaging and as PTAs.^46^ Organic dyes can be rapidly cleared from the body, preventing high toxicity, yet this can limit the duration of the treatment. Such dyes also have limited tumor selectivity, and generally poor stability in aqueous mediums.^57,79,80^ To address these limitations, organic dyes have been commonly associated with other nano-sized structures, such as micelles or liposomes, in an attempt to increase their stability and tumor selectivity.^80^ Examples of organic dyes used as nano-PTAs include indocyanine green (ICG),^55,81,82^ methylene blue (MB),^53,83^ and IR780.^84–86^

### 2.2 Magnetic hyperthermia

In MH, magnetic nanoparticles (MNPs) are used to transduce magnetic energy produced by an AMF into heat.^34,87,88^ The inability of the magnetic moments of the MNPs to follow the AMF totally in phase leads to hysteresis and heat generation. This inability may be due to the physical rotation of the MNPs (Brown mechanism), to the rotation of the magnetic moment as a single moment across an energy barrier provided by the crystal lattice (Neél mechanism), or to rearrangement of the orientation of the magnetic moments in the case of multi-domain larger MNPs. The heat generated by the MNPs is dependent on the frequency of the applied magnetic field, the size and morphology of MNPs, and the biological properties of the tissue.^87,89–91^ The amount of heat dissipated per unit mass of MNPs is commonly quantified by the specific absorption rate (SAR), which indicates MNPs heat generation efficiency upon the application of a magnetic stimulus.^92,93^

The efficiency of the MNPs in MH depends on their coating, size, morphology, and magnetic properties (*e.g.*, saturation magnetization (*M*_s_), magnetic susceptibility, and magnetocrystalline anisotropy).^34,88,94,95^ The size and morphology of the MNPs are key features to be considered due to their influence on cellular uptake, which will be further discussed.^96^ Moreover, the size of the MNPs will influence the mechanism responsible for generating heat within the tumor: hysteresis losses due to domain rotation/reconfiguration are more significant in multi-domain NPs, and Brownian and Néel relaxation losses are dominant in single-domain NPs.^90,97,98^ The size distribution of MNPs should be uniform to contribute to a homogeneous distribution of heat, and the MNPs should be well dispersed in small concentrations.^87,88,90^ Usually, larger particles have a higher *M*_s_, which is proportional to the heating efficiency of the MNPs. So, a compromise between the size of the NPs must be found to maximize their heating capacity and cellular uptake.^87,99,100^ Besides being important to guarantee a high *M*_s_ and magnetic susceptibility, MNPs should have a suitable volume and magnetocrystalline anisotropy, whose product is the energy required to change the orientation of the magnetic moment of the particles, to match the characteristic relaxation time and the AMF frequency, aiming to maximize the heating efficiency.^88,101,102^

Functionalization of MNPs with hydrophilic and low toxic materials is usually performed to increase both the dispersibility of NPs in water and colloidal stability.^94^ Moreover, it improves biocompatibility and tumor target selectivity, prevents MNPs from agglomerating, and increases the NPs' circulation time.^87,95,103,104^ The material of the surface coating, its thickness, and the size of the core influence the heating efficiency of the final particle. Usually, a thinner coating and a larger core size contribute to a higher SAR, while a thicker coating and a smaller core size contribute to a lower SAR by inhibiting Brownian relaxation. Moreover, MNPs with a low dispersibility behavior require a thicker coating to improve their stability and prevent aggregation.^88,105^ So, a compromise between the thickness of the coating and the size of the core must be found to maximize the SAR, dispersibility, and colloidal stability in aqueous medium.^88^ Coating MNPs with appropriate materials such as poly(lactic-*co*-glycolic acid) (PLGA), polyvinyl alcohol (PVA), poly(vinylpyrrolidone) (PVP), polyethylene glycol (PEG), or dextran has been shown to improve NPs-cell interactions and also prevent toxic side-effects.^106,107^ Lipid-based nanomaterials, such as liposomes^108,109^ or niosomes,^110,111^ have also been used as coatings for MNPs due to their biocompatibility, flexible design, and surface modification capacity. Both polymeric and lipid coatings allow for a controlled release of the inner NPs/drugs upon the use of different stimuli (*e.g.*, temperature, pH, light, magnetic or electric fields, *etc.*), which is advantageous, for example, for target drug delivery and for MRI contrast.^112,113^ Recently, hybrid systems consisting of polymer and lipid coatings have emerged as promising approaches capable of improving NPs’ stability, biocompatibility, and drug release kinetics.^114,115^

Superparamagnetic iron oxide NPs (SPIONs) have been the most used for MH as they show valuable size-dependent magnetic properties, suitable biocompatibility, reduced toxicity, high surface-to-volume ratio, proper stability in aqueous suspension, can be easily functionalized, and are FDA approved.^116–118^ Moreover, these particles have been shown to have the potential to induce heat by optical stimulation, which, in combination with MH, can contribute for increasing the efficiency of candidate anti-cancer treatments.^119^Table 2 summarizes the most recent studies exploring MNPs for hyperthermic nanomedicines screening in flat 2D *in vitro* models. The use of MNPs has several advantages since, in addition to being used as heat sources, they can also be used as contrast agents, non-invasive temperature thermometers, or for complementary targeted drug delivery.^120–122^ However, it is still a challenge to guarantee that the MNPs are homogeneously distributed in the target region, achieve uniform heating, and to ensure that the magnetic properties of the NPs/administered concentration are adequate to promote an effective outcome.^119,123^

**Table 2:** Examples reporting the performance of MNPs for 2D *in vitro* MH

Heating agent			Heating conditions	SAR^c^ (W g^−1^)	*M*_s_ (emu g^−1^)	2D tumor model	Dose (μg mL^−1^)	*T* reached (°C)	Cell death (%) (time after treatment, technique)	Ref.
Core	Coating	Size w/coating (nm)								
Fe_3_O_4_	Aminosi-lane	110, DLS	557 kHz, 300 Gauss, ∼24 kA m^−1^, 40 min	338	N.D.	C6	100^b^	44	∼70 (N.D.; FC)	126
Fe_3_O_4_	CA	46, TEM	60 kA m^−1^, 30 min	2380^b^ (325 μg mL^−1^ b, 29 kA m^−1^)	83	A549	46^b^	43.6	∼20^a^	127
							85.5^b^	45.95	∼80^a^	
							177.5^b^	49.67	∼100% (24 h, AB)	
Fe_3_O_4_	PCL	21, TEM	318 kHz, ∼32 kA m^−1^, 15 min	153 (10^3^ μg mL^−1^)	64	HepG2	100	43.2	∼39	128
			313 kHz, ∼47 kA m^−1^, 15 min	201 (10^3^ μg mL^−1^)				46.1	∼60% (24 h, MTT)	
Fe_3_O_4_	PVP	145, DLS	∼333 kHz, 170 Oe, 15 min	160 (10^4^ μg mL^−1^)	72	MDA-MB-231	500	42.5	75 (24 h, MTT)	129
CoMn–Fe_2_O_4_	PEG–PCL	∼79, DLS	420 kHz, ∼27 kA m^−1^, 30 min	1237 (w/coating)	93 (w/o coating)	ES-2	50	55^a^	∼99 (48, calcein AM)	130
GO–Fe_3_O_4_	—	20, XRD	236 kHz, ∼4 kA m^−1^, 10 min	70^a^	50^a^	HeLa	1000	N.D.	40 (2 h, MTT)	131
MnFe_2_O_4_	—	31, TEM	765 kHz, 300 Oe, (1) 4–6 min followed by (2) 18–23 min after 2 days	∼300^a^	∼70	Saos-2	250	(1) ∼45^a^	(1) 25^a^; (2) 75^a^	132
							500	(2) ∼43^a^-for both doses	(1) 30^a^; (2) 90^a^ (N.D.)	

a Value extracted from graphs.

b Dose of Fe.

c The conditions that are not indicated are the same as the heating conditions; N.D. – non described; FC: flow cytometry; AB: Alamar Blue; CA: citric acid; PCL: polycaprolactone; PVP: polyvinylpyrrolidone; GO: graphene oxide.

MH and PTT are complementary, in the sense that while PTT is more suitable for surface and near-surface applications, MH is applicable in deep tissues, since the penetration of the AMF exceeds the penetration depth of light.^94,124,125^ Consequently, MH can be used to trigger hyperthermia in deeper tumors than PTT.^87^

When designing nanomedicines for hyperthermic applications, it is important to consider the factors that affect their penetration in tumors that is influenced by NPs’ properties (*e.g.*, size, morphology, or surface chemistry/functionalization), and by physical and biological barriers (*e.g.*, uptake by the immune system, shear stress under circulation, renal filtration, interstitial fluid pressure, or tumor desmoplasia).^133,134^ In general, NPs bigger than 200 nm are easily accumulated in the liver and spleen, and NPs smaller than 6 nm are usually filtered by the kidney, not being able to accumulate in the target site.^95,135^ Moreover, coating the NPs with neutral polymers generally reduces clearance mediated by the immune system.^133^ Since some tumor types have a leaky vasculature with increased permeability and poor lymphatic drainage, nano-sized agents are more likely to be passively internalized and retained by the tumor after systemic administration, which is referred to as the enhanced permeability and retention (EPR) effect.^95,136^ In fact, the internalization of nano-agents as drug carriers in tumors can exhibit over a 10-fold increase in effectiveness compared to free-drugs, even though only 10 to 15% of injected NPs successfully accumulate within the tumor.^137^ Approaches aiming to improve the accumulation and retention of NPs in tumors include, for example, the use of ligands or antibodies to bind with specific malignant cell receptors to promote a targeted delivery.^138–140^ Regardless of their physicochemical features, the preclinical validation of such systems is a requirement before they are considered for clinical applications.

To date, the preclinical performance and validation of such engineered nano-heating agents for localized heat generation has been mainly performed in 2D cell monolayers and/or mice models.^141,142^ Recently, the use of 3D *in vitro* models in the design and validation stages of hyperthermic nanomedicines has been rapidly emerging as an alternative strategy, owing to its potential to overcome the issues of 2D cultures, for significantly reduce animal model usage, and for accelerate the identification of top performing nano-heating agents.

To fully explore the potential of 3D *in vitro* tumor models for evaluating the performance of hyperthermic nanomedicines, it is important to discuss the relevance of the third dimension in what relates to NPs’ penetration and distribution in the complex TME, as well as heat transfer mechanisms, that are otherwise more difficult to be modeled in 2D cell cultures.

## 3 *In vitro* tumor models for nanomedicines screening

### 3.1. Mimicking the tumor microenvironment (TME): 2D *versus* 3D models

The complexity of tumors and their interactions with the surrounding microenvironment has prompted researchers to develop more advanced and sophisticated 3D tumor models that better recapitulate different tumor hallmarks in a preclinical setting when compared to the limited 2D flat cell monolayers methodologies.^143^ Owing to the inherent spatial differences, 2D and 3D tumor models recapitulate the TME components and its biological hallmarks differently in an *in vitro* setting.

In the native TME, the tri-dimensional existence of the surrounding stroma plays an important role in tumor progression, invasion, and metastasis, for example by producing enzymes to degrade the ECM, by supplying biomolecular cues to promote cancer cell growth and angiogenesis, by suppressing the immune response, or by recruiting healthy cells.^144–146^ The stroma is comprised by the ECM and other cell types, such as endothelial cells, cancer-associated fibroblasts (CAFs), mesenchymal supporting cells, cells of the vascular/lymphatic system, and immune system cells.^147–149^ In malignant tissues, the ECM undergoes significant alterations resulting in *de novo* deposition of ECM, where proteins (*e.g.*, collagen, fibronectin, laminin, *etc.*) are upregulated and an enzyme-mediated (*e.g.*, lysyl oxidase) stiffness increase occurs, which represents a physical barrier to therapies.^27,150,151^ Each cancer-associated cell type has its own function on tumor progression, making it important to consider the rationale addition of these living units in the design stage of a 3D *in vitro* tumor model.^144^ For example, fibroblasts contribute to ECM *de novo* deposition and remodeling of ECM proteins, facilitating the invasion of cancer cells into neighboring tissues, while immune cells can also have tumor promoting capacity after being recruited by other cancer cells.^149,152^

In conventional 2D platforms, cells grow as monolayers in adherent conditions. This results in a flat and homogeneous environment that deprives cell–cell and cell–ECM interactions and leads to changes in cellular morphology and function. Moreover, 2D models were shown to modify cellular polarity, morphology, secretion, gene expression, and signaling. These models generally only focus on one cell type (monotypic), making it difficult to co-culture different cellular components, which often neglects the essential contribution of stromal components to tumor development. 3D tumor models provide a far more realistic volumetric architectural rearrangement and cellular representation of the TME by allowing the inclusion of stromal components and enabling to easily recapitulate cellular diversity and heterogeneity.^14,26,141,153^

Since cancer cells are highly proliferative, the angiogenic process (*i.e.*, formation of vasculature) cannot generally keep pace, leading to the disorganized formation of leaky and branch vessels with irregular sizes and increased permeability, in some cancers.^154^ This, in turn, results in an increase in the interstitial pressure. Consequently, the inner cells of the tumor mass become deprived of nutrients and oxygen, leading to the formation of a hypoxic and acidic environment with gradients of oxygen, nutrients, and metabolites.^152,155,156^ 2D *in vitro* testing platforms, where cells are cultured in an air–liquid interface, are unable to mimic these gradients or allow to recapitulate the formation of vessel-like structures, elements that are essential to better understand tumor behavior.^23^

The hypoxic microenvironment leads cancer cells to adapt their metabolism to promote survival, invasion, and metastasis. These adaptative changes are further supported by proteomic and genomic alterations.^157^ Interactions between the stroma and cancer cells can also drive cancer progression by secreting growth factors and signaling molecules (*e.g.*, transforming growth factor-β (TGF-β), stromal-derived factor (SDF-1), *etc.*).^158^ Hypoxia also promotes the epithelial–mesenchymal transition (EMT) that plays an important role in metastasis. These mechanisms are predominantly driven by the TME-tumor interactions, which are highly challenging to mimic in 2D models.^159–162^ Moreover, the oxygen-deprived environment contributes to an heightened resistance against radiation and constrains drug penetration.^150,163,164^ Additionally, cancer stem cells (CSCs) play a prominent role in tumor resistance and metastasis, as they can self-renew and regenerate tumor populations after treatment.^150,165,166^ 3D models facilitate the modulation of CSCs population, which is more challenging to be modeled in standard 2D models, allowing for a better recapitulation of tumorigenesis and resistance mechanisms.^167,168^

In essence, 3D tumor models represent a testing platform that more closely resembles the architectural complexity, cellular interactions, environmental conditions, and underlying mechanisms that occur in tumors. By employing these models, it is possible to provide a more accurate assessment of nanomedicine penetration and efficiency, allowing to evaluate different coatings and targeting approaches. Consequently, 3D models can provide data that is not attainable in 2D and contribute to accelerate the validation of hyperthermic nanomedicines for clinical practice.

### 3.2 3D tumor models for preclinical screening of therapeutics

**Fig. 2:**
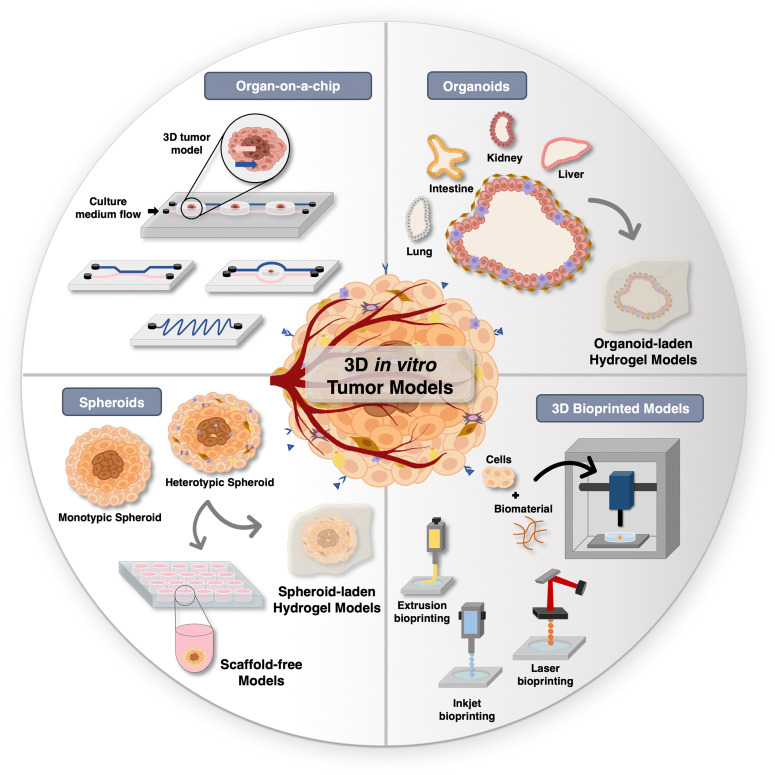
Overview of *in vitro* tumor models: Schematic of diverse 3D tumor models for hyperthermic nanomedicines screening.

In the pursuit of better alternatives to conventional 2D monolayered models, researchers have been focusing on developing different types of 3D tumor models, with a particular emphasis on spheroids, organoids, organ-on-chip platforms, and 3D bioprinted models (Fig. 2), all of which are addressed in detail in the following section.

#### 3.2.1 Spheroids

Spheroids are the most commonly used 3D *in vitro* models and consist of spheroidal, randomly self-assembled cell-rich structures.^169–172^ These models can be comprised of only one cell type (monotypic) or several cell types (heterotypic).^173,174^ Heterotypic spheroids can be bioengineered with tunable cell density/size, and have been used to screen different types of candidate therapeutics.^174,175^ Since heterotypic spheroids better mimic the cellular complexity of native tumors, these are considered more clinically relevant models. Heterotypic spheroids have been used to study the influence of different cell types (including stromal cells) on tumor progression, gene and protein expression patterns, to assess drug and nanoparticles penetration, as well as chemotherapeutics response.^176–179^ However, the generation of compact spheroids may not be achievable with all cell types, and spheroid morphology is generally dependent on the cell type and the culture conditions that are employed.^24,169^ Persistent challenges include standardization of spheroids production techniques and modeling vascular and immune system elements of tumors in these models.^180^

Spheroids generation methods can be generally categorized as scaffold-based or scaffold-free techniques. Scaffold-free techniques leverage the inherent capacity of cells to self-aggregate to form spheroidal structures and, subsequently, to potentially secrete their own ECM over time in culture. The simplicity and cost-effectiveness of these models render them highly sought-after by researchers. On the other hand, scaffold-based methods involve the incorporation of malignant and/or stromal cells within tailored hydrogels designed to mimic the ECM and to provide structural support, adhesion sites, as well as biomolecular stimulation.^23,169,181^

#### 3.2.2 Organoids

Organoids can be produced from embryonic or induced pluripotent stem cells embedded in a hydrogel matrix. Organoids resemble the heterotypic cellular composition, organization, genetic traits, and biofunctionality of the human organ of origin, being capable of self-organizing and evolving in culture over time. These models allow a co-culture of multiple cell types and can be used to model the TME and the cancer-stromal interplay.^171,172,182^ Organoids can be cryopreserved and used for personalized medicine, enabling researchers to explore patient-specific approaches.^182–184^ In the context of oncology, organoids can be derived from healthy or tumor tissues, enabling the evaluation of drugs performance in these different scenarios, under controlled conditions.^182^ Despite their higher correlation with the native tissue, organoids generation is generally a cumbersome and expensive process, and these models generally still lack a comprehensive representation of the vasculature, biomolecular gradients, and immune system cells present in human tumors. The widespread use of organoids encompasses several challenges particularly regarding reproducibility, standardization, cost-effectiveness, and scalability, yet they are one of the most biomimetic models of human disease available to date.^182,184,185^

#### 3.2.3 Organ-on-a-chip platforms

Organ-on-chip platforms, also termed microphysiological systems, have been widely used to recapitulate human diseases, including cancer on microfluidic chips under dynamic flow conditions.^186,187^ These models are generally designed to reconstitute the structural, microenvironmental, and functional complexity of human organs, as well as modulate the mechanical properties at different fluid-related shear stress, the nutrient supply, waste removal events, and the dynamic interactions occurring between cells and the flowing culture medium to recapitulate human pathophysiology.^187–189^ This is a relatively low-cost and reproducible methodology that enables the manipulation of parameters of the TME in the space and time domains. Additionally, some microfluidic platforms enable a real-time and quantitative assessment of tumor development and progression.^27,186–188,190^ Gathering on their modularity and versatility, researchers have been exploring tumor-on-a-chip models to integrate microvascular systems and their fluid dynamics. Since virtually any spheroid or organoid model can be included in microfluidics platforms, these microphysiological platforms are considered highly advanced and more biomimetic.^191^ Their development holds great promise for evaluating drug or nanoparticles penetration and their biological performance under dynamic conditions.^191–194^

From a practical perspective, organ-on-a-chip platforms are often fabricated by using poly(dimethylsiloxane) (PDMS), a biocompatible silicon-based polymer. In addition, hydrogels capable of closely mimicking ECM properties, such as Gelatin methacryloyl (GelMA), can be included in the microfluidic chambers to provide support to cancer cells/spheroids or organoids.^27,195,196^ Tumor-on-a-chip models have been widely used in cell biology, single-cell studies, drug discovery, genetic assays, intracellular signaling, toxicology studies, and tissue engineering.^197,198^ Breast,^199,200^ colorectal,^201,202^ and brain^203,204^ tumor-on-a-chip models have already been assembled.

#### 3.2.4 3D bioprinting

In recent years, 3D bioprinting has rapidly emerged as a valuable technique for generating customized and larger scale (mm up to cm scale) 3D tumor models. Extrusion bioprinting is one of the most widely used 3D bioprinting methods and involves the sequential deposition of layers of a given bioink (*i.e.*, polymer + cells) to construct a tailored 3D tumor model that can comprise cancer and stromal cells. This technique allows a precise volumetric placement of cells, bioactive factors, and biomaterials to mimic the TME, having the ability to create geometrically complex scaffolds with low cost and reproducibility. Biomimicry of tumor heterogeneity can be achieved by printing different cell types and ECM components. Furthermore, the dimension and geometry of the scaffold can be adapted, and the underlying ECM mimetic biomaterial properties can be tuned, enabling the creation of personalized microarchitectures with biomimetic capabilities.^189,205,206^ Other methodologies explored to generate 3D tumor models by bioprinting include laser, droplet, or dynamic light projection (DLP).^207,208^ 3D bioprinting techniques face significant challenges related to bioink formulation, reproducibility, and standardization, maintenance of the integrity of the printed components, preserving cell viability, and optimizing the rheological or viscoelastic properties of the biomaterial inks used for assembling the tumor models.^189,207^

3D bioprinting has been used to assemble spheroids, organoids, and tumor-on-a-chip models. Moreover, attempts have been made to implement vasculature in tumor models with this technique, particularly within microfluidic platforms.^209,210^ Adding to this, 3D bioprinting can be helpful to generate customizable 3D *in vitro* models with a user defined size that better mimics that of the human scale,^211^ which is advantageous for pre-clinical imaging techniques, such as magnetic resonance imaging (MRI), positron emission tomography (PET), or computed tomography (CT), that have limitations in sample size due to their spatial resolution.^212^

In addition to the discussed differences between 2D and 3D *in vitro* tumor models, the latter may also offer the ability to manipulate the non-cellular microenvironment through the encapsulation of cells in ECM-mimetic hydrogels. These, so-termed scaffold-based 3D *in vitro* models, are highly valuable to study the influence of the biophysical and biomechanical properties of the ECM, particularly through the modulation of various parameters that exist in the tumors matrix (*e.g.*, establishment of chemical gradients, shifts in stiffness/viscoelasticity, evolving biomolecular composition, mass transfer phenomena), thus enabling to more accurately mimic *in vivo* conditions.^213,214^ Furthermore, these scaffold-based *in vitro* models provide the possibility to model the influence of each parameter in different stages of tumor development.^213^ In this framework, recent endeavors have focused on bioengineering ECM-mimetic hydrogel 3D tumor models with on-demand tailored mechanical properties to study tumor growth,^215^ migration/invasion,^175,216,217^ or metastasis.^218^ Adding to this, cutting-edge bioengineering methods, such as 3D bioprinting, offer the ability not only to generate *in vitro* models with controlled mechanical properties but also with a physiomimetic architecture of the microenvironment.^214,219^ In a complementary approach, the integration of 3D bioprinting with microfluidic technology has been proven to be crucial in integrating mechanical cues within 3D *in vitro* models, encompassing shear flow, gradients, and mechanical stimulus. These cues, in turn, can influence cellular signals, cell adhesion molecules, cytoskeleton dynamics, and activation of membrane transporters and ion channels.^220,221^ By manipulating these properties in 3D *in vitro* models, researchers can create a more realistic and tailored model to better predict cells' response to treatments.

### 3.3 Heat response and transfer mechanisms: probing dimensional impacts on heat response in 2D and 3D models

During stress, such as oxidative, pH, hypoxic, heat, or radiation, an overexpression of heat shock proteins (Hsp) is initiated to promote cell survival, especially involving Hsp70 and Hsp90. This includes binding to denatured proteins, preventing incorrect aggregations, assisting protein assembly, secretion, and degradation, as well as transporting those proteins through membranes.^14,222^ Hsp are mainly regulated by the heat shock factor 1 (Hsf1), which normally resides in the cytoplasm in an inactive state. In response to a stress situation, Hsf1 enters the nucleus and initiates the transcription process, resulting in the production of Hsp. Once the stress is removed, Hsf1 returns to its inactive state in the cytoplasm.^19,223^ This response enables cells to maintain their functionality, evade the signals that trigger cell death, and mitigate the effects of drugs, leading to the development of resistance to treatments.^224^ Consequently, researchers have directed their efforts towards discovering strategies to target Hsp with inhibitors, aiming to increase cancer cells death upon treatment.^19,225^ Moreover, hyperthermia has shown to promote the infiltration of immune cells in tumors, enhancing immune response and modulating the TME. This dynamic interplay can improve tumor cell recognition and destruction by the immune system.^6,226^

The response of cells to stress is influenced by the conditions of the culture. In 2D models, cells are simultaneously and uniformly placed under similar conditions, leading to a higher rate of cell death when compared to 3D cultures. This is primarily due to the additional architectural, cellular, and environmental components of 3D models.^14,227^ Such testing platforms are highly valuable to better understand and predict the performance of candidate hyperthermia treatments.

The temperature in tissues is the result of a balance of factors, including ambient temperature, heat generation as a result of metabolic activity, and heat transfer from hot to cold regions of the body by conduction and convection.^228,229^ It is worth noticing that convection is quite more efficient in transporting heat.^230^ According to a study, the size and shape of NPs, blood flow, and vessel geometry can all impact the distribution of NPs inside tumors, which in turn can affect the temperature distribution.^231^ Moreover, nanosized particles are more efficient in penetrating deeper into tumors but have high elimination rates.^231,232^ Another study demonstrated that the rate of heat generation and its impact on surrounding tissues are influenced by the infusion rate, blood flow, and distribution of nanoparticles within the tumor. A lower infusion rate was shown to be more successful in raising the concentration of NPs in the TME, causing less heat to dissipate to the surrounding tissue which can damage it.^233^ Heat dissipation to the surrounding tissues is another important consideration, as it can have an impact on the safety and effectiveness of hyperthermia therapy. As the volume of the tumor grows, less heat is dissipated, resulting in a greater temperature differential between the tumor and the surrounding media, allowing for the selective heating of the tumor, as more heat is carried away by perfusion on the normal tissues.^234–237^ Due to all the complex factors that have to be considered for hyperthermia, advanced techniques and technologies for heat transfer modeling have been developed. The heat balance of the tissues was first described by the bioheat equation proposed by Pennes’, which is now a standard model for studying the temperature distribution in tissues. This model states that the heat stored in the tissue is equal to the balance between the heat generated by metabolic activity and the heat dissipated by conduction and convection.^238,239^ Beyond the Pennes’ bioheat model, others have been developed, such as the local thermal equilibrium (LTE) and local thermal non-equilibrium (LTNE) equations, and dual-phase-lag bioheat model.^239,240^ The LTE and LTNE models consider the existence of a porous medium that consists of a solid matrix (*i.e.*, tissue) and blood vessels that are in thermal equilibrium or non-equilibrium, respectively. The LTNE model represents a more realistic situation since it considers a temperature gradient between both phases.^239–242^ Pennes’, LTE, and LTNE models consider that the blood velocity is infinite (based on Fourier's law), meaning that when applying heat, the temperature of a tissue changes immediately, which does not happen in non-homogeneous tissues. The dual-phase-lag (DPL) bioheat model considers that the temperature and heat flux have a lag time. Consequently, this model focuses on the micro-structural interactions and considers that the tissues and blood can have different temperatures.^239,240,243–245^Table 3 summarizes the main advantages and disadvantages of each model, as well as examples of the application of these models to predict heat distribution in tumors.

**Table 3:** Advantages and disadvantages of the bioheat models used to predict heat distribution in tumors, compiled from references^239–245,258,259^

Model	Description	Advantages	Disadvantages	Application of the model to predict heat distribution in tumors
Pennes’ bioheat equation	The heat stored in the tissue is equal to the balance between the heat generated by metabolic activity and the heat dissipated by conduction and convection	Simplicity; consideration of blood perfusion	Assumes a homogeneous and isotropic tissue; constant blood perfusion; restricted spatial variability; do not consider the vascular geometry	247, 254, 255 and 257
Local thermal equilibrium equation	Considers the existence of a porous media that consists on a solid matrix (*i.e.*, tissue) and blood vessels that are in thermal equilibrium	Simplicity; can consider blood flow direction	Assumes that the temperature of the blood and tissue is the same; it can only be applied in scenarios with limited vessels diameter and blood velocities	260
Local thermal non-equilibrium equation	Considers the existence of a porous media that consists of a solid matrix (*i.e.*, tissue) and blood vessels that are in thermal non-equilibrium	Consider the temperature of tissues and blood; can consider blood flow direction; improved model compared to the one-equation model	Complexity; detailed information about thermal properties of tissues is required	247 and 260–262
Dual-phase-lag bioheat equation	Considers that the there is a lag time in the temperature and heat flux	Consider the temperature of tissues and blood; consider micro-structural interactions effects	Complexity; detailed information about thermal properties of tissues is required	263–266

**Fig. 3:**
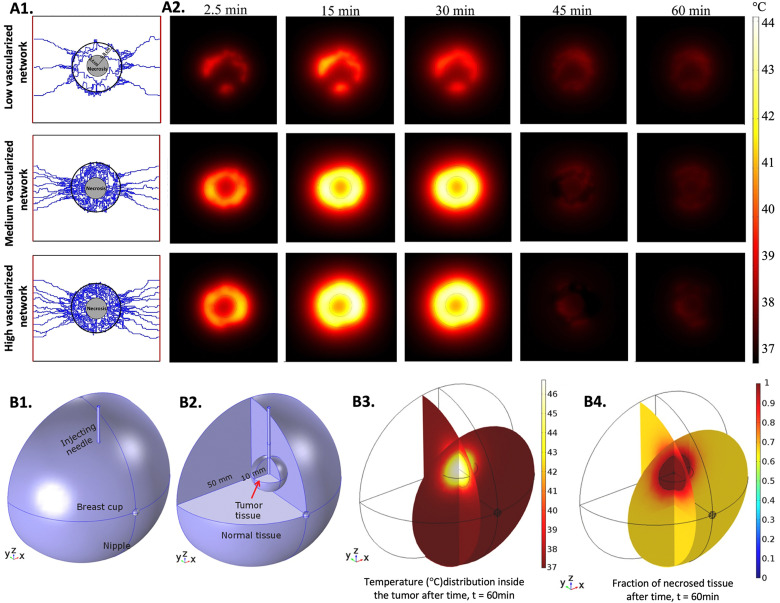
Simulations of heat distribution in 3D tumor models: (A1) three capillary models with low, medium, and high vascularization used to perform the numerical studies, (A2) computational estimation of temperature distribution for three different tumor vasculature densities at five time points. The MNPs were injected at constant rate for 30 minutes. Reprinted from ref. 254, Copyright (2022), with permission from Elsevier and (B1) designed model representing the breast cup with the injecting needle, (B2) designed model enhancing the tumor and the normal tissue, (B3) 3D simulation of the temperature distribution in the breast cup and (B4) 3D simulation of the necrotic tissue. Reprinted from ref. 255, Copyright (2020), with permission from Elsevier.

When predicting hyperthermia effects in 3D *in vitro* tumor models, the choice of the bioheat equation depends on the specific characteristics of the tissue. For instance, a study concluded that the DPL model can better predict heat transfer in tissues with larger blood diameters, but when considering tissues with micro-capillaries, the Pennes’ model and the DPL model had similar performances.^246^ In another study, the performance of the LTNE model with variable porosity and the Pennes’ bioheat model with variable perfusion was compared when simulating thermal ablation in tumor tissue, and it was shown that the LTNE model was more accurate in predicting heat distribution after comparing the results with *in vivo* experiments.^247^ Although, due to its simplicity, the Pennes’ bioheat model has been the most used to study heat transfer in biological tissues.^248^ In a balance between simplicity and accuracy, the Pennes’ bioheat equation has undergone some modifications over the years, aiming to predict heat distribution in tumors as close as possible to an *in vivo* situation. Different parameters, such as the direction of the blood flux, spatial and temporal variations of the velocity of the blood, size, geometry, and density of the vasculature, and the distribution of nanomedicines, have been considered.^231,249–253^Fig. 3 shows the use of advanced tumor models to better understand heat distribution, particularly by varying the density of tumor vasculature (Fig. 3A1 and A2),^254^ and by using Fe_3_O_4_ MNPs for MH (Fig. 3B1–B4).^255^ Even though recent studies have been providing important information, it is difficult to resemble a perfect *in vivo* situation due to the complex and unpredicted variables, such as tumor size or the geometry and density of the vasculature, that have a huge influence on how the NPs and heat are distributed. Moreover, it is difficult to confirm the veracity of the obtained results due to the lack of experiments performed in clinical settings.^254^ Currently, researchers are dedicated to improving mechanisms to predict heat delivery in tissues by introducing variables such as the size and morphology of the NPs.^231^ Significant advances in this direction are expected in the upcoming years, especially considering that 3D hyperthermic simulations are an important tool to accelerate the clinical translation of nano-hyperthermia.

Numerical simulation methods are a valuable resource for hyperthermia treatment planning. These methods can help to predict how the chosen treatment for the specific size and location of the tumor can affect both tumor and healthy tissue, and to adapt the heat and heating agents’ doses needed for personalized treatment. Moreover, by comparing the outcomes of these methods with the ones obtained from 3D *in vitro* models, it is possible to validate these theoretical techniques for modulating the tumor response to hyperthermia. These improvements, combined with the increasing computational power and the advent of artificial intelligence (AI) algorithms for simulation, are envisioned to become a major part of the preclinical optimization/validation process of hyperthermia therapies and of the research to predict their effect on healthy tissues.^239,256,257^

## 4 Advances in preclinical screening of hyperthermic nanomedicines in 3D tumor models

The development of 3D tumor models has revolutionized the field of cancer research and encouraged their application for the screening of hyperthermic nanomedicines.^14^ By using these models, it is expected to have a deeper understanding of the interactions between hyperthermic nanomedicines and cancer cells, as well as the influence of the TME on drug delivery, nanomedicines distribution in the tumor, and therapeutic response. With a clear understanding of these mechanisms, researchers can optimize treatment strategies and develop more clinically relevant hyperthermic approaches for cancer therapy.^14,267,268^

This chapter aims to provide an overview of the recent advances in the preclinical screening of hyperthermic nanomedicines using 3D tumor models, with a prominent role in PTT, MH, and hyperthermia combined with conventional therapies. By examining the cutting-edge research in this field, it is expected to provide valuable insight into the potential of 3D tumor models as powerful tools for preclinical screening, allowing the identification of hyperthermic nanomedicines with optimal therapeutic efficacy and safety profiles.

### 4.1 3D tumor models for photothermal nanomedicines screening

**Fig. 4:**
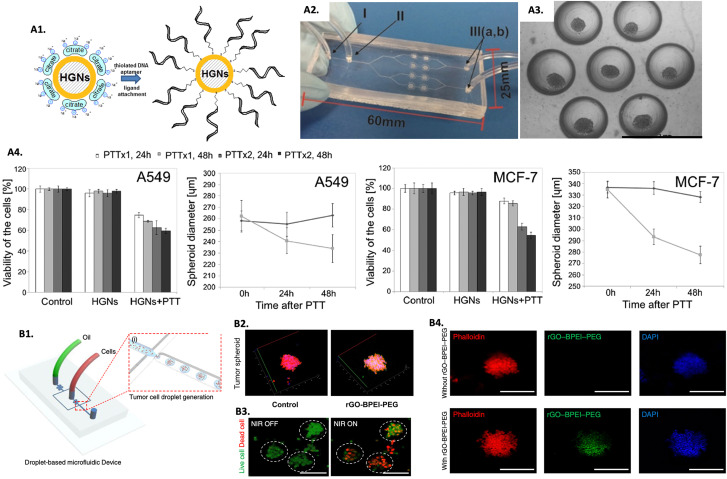
Screening PTT nanomedicines in 3D *in vitro* tumor models for PTT: (A1) scheme showing the modification of HGNs with the anti-MUC1 aptamer, (A2) microfluidic system used to implement the spheroids (I, III – inlets/outlets and II – vent hole), (A3) image of a chamber of the microsystem containing spheroids, and (A4) spheroids viability and diameter submitted to one (PTTx1) and two (PTTx2) NIR doses, after 24 and 48 hours. Reprinted with permission from ref. 272, Copyright (2019), with permission from Elsevier; (B1) scheme of the generation of 3D tumor spheroids using the drop-based microfluidic device, (B2) Z-stack confocal laser scanning microscopy image of 3D tumor spheroids treated with rGO-BPEI-PEG (scale bar = 100 μm), (B3) live/dead images of brain tumor spheroids before and after PTT (scale bar = 100 μm), and (B4) fluorescence microscopy images of brain tumor spheroids with and without rGO-BPEI-PEG internalized, reprinted with permission from ref. 274, Creative Commons (CC) License 4.0 (https://creativecommons.org/licenses/by/4.0/).

The emergence of 3D *in vitro* tumor models represents a relatively recent development, and as such, numerous studies are still dedicated to understanding the differences in the hyperthermic response between 2D and 3D models. Comparative studies evaluating the efficacy of hyperthermic nanomedicines within monolayers and tumor spheroids revealed that 3D models are more resistant to the treatment.^269,270^ Moreover, an additional study showed that 2D monolayers needed less NPs incubation time and had more efficiency in binding the NPs to cells than 3D models, enhancing the importance of replacing 2D with 3D models for treatment parameters optimization, aiming to obtain more relevant outcomes.^271^ More complex models using microfluidic platforms to grow spheroids have been focused on understanding the penetration, distribution, and effectiveness of hyperthermic nanomedicines through more complex 3D models. As an example, a microfluid system consisting of PDMS was used to grow multicellular tumor spheroids to evaluate the PTT performance of hollow Au nanoshells modified with an anti-MUC1 aptamer (HGNs@anti-MUC1) (Fig. 4A1–A3).^272^ Human lung epithelial carcinoma cells (A549) and human breast adenocarcinoma cells (MCF-7) were used to assemble the tumor spheroids. The tested nano-PTA showed to be efficient in reducing the viability and size of tumor spheroids after irradiation, which was more pronounced with a double dose of irradiation with a 1 hour interval from the first one (Fig. 4A4). The study also tested the nano-PTA in spheroids of non-tumorous cell lines, and besides proving that the internalization of the nanomedicine was less effective compared to tumor spheroids, it was also observed that under the same treatment conditions, non-tumorous spheroids exhibited less pronounced effects, assuming the efficiency of HGNs@anti-MUC1 to specifically treat tumors. Furthermore, spheroids from different cell lines had different treatment outcomes, reinforcing the fact that the response to hyperthermia is tissue-dependent.^272^

Treating brain tumors presents significant challenges as it involves the risk of damaging surrounding healthy tissue, which can lead to serious complications and low survival rates. The non-invasive nature of hyperthermia, its potential for selective tumor treatment, and the challenges posed by the complexity of the brain TME and the blood–brain barrier have encouraged the exploration of this therapy in 3D brain tumor spheroids.^34,273^ As an example, U87MG glioblastoma cells spheroids generated in a PDMS microfluidic device (Fig. 4B1) were used as a screening platform to evaluate a reduced graphene oxide-branched polyethyleneimine-polyethylene glycol (rGO-BPEI-PEG) nanocomposite for PTT.^274^ After confirming the efficient uptake of the nano-PTAs in the brain tumor spheroids (Fig. 4B2 and B4), a significant decrease in cell viability was observed after PTT (Fig. 4B3).^274^ In another study, macrophages were used as vehicles to transport gold–silica nanoshells (AuNS) and gold nanorods (AuNR) to human glioma (ACBT) spheroids.^275^ A better uptake was observed with the AuNR due to their shape and smaller size, however, the AuNS presented more efficiency than the AuNR in reducing the spheroids volume after PTT, a difference that increased with the increase in intensity of irradiation. The authors explained these findings by the larger cross-sectional area of the AuNS that resulted in greater efficiency in transforming light into heat.^275^

A recent study using 4T1 tumorspheres showed that cypate (Cy)-loaded hyaluronic acid (HA)-black phosphorus nanosheets (Cy@HBPN) were able to significantly suppress the growth of the 3D models and inhibit their regenerative potential.^276^ After proving the *in vitro* potential, a mouse xenograft tumor model administered with Cy@HBPN showed to completely inhibit tumor growth after laser irradiation, validating the *in vitro* results.^276^ In another study, organoids were explored as testing platforms to access black phosphorus quantum dots into exome vector nanospheres (BEs) hyperthermic efficiency.^277^ BEs were able to internalize cells, inhibit tumor progression, and suppress angiogenesis. Further *in vivo* assessment in a nude mouse model bearing a subcutaneous bladder tumor confirmed the ability of BEs to inhibit tumor growth and recurrence, revealing BEs good photothermal and targeting capability.^277^

Beyond demonstrating the feasibility of using PTT to replace traditional methods by being suited to perform treatment in tumors localized in deeper and sensitive parts of the body, such as the brain, the reported studies showed that 3D *in vitro* platforms are important tools to predict the ability of nano-PTAs on penetrating and accumulating in the tumor, to understand what NPs physicochemical properties can be modulated (*e.g.*, surface, size, shape modifications, or targeting ability) to reach maximum retention of the agents at the tumor site and improve the outcome of the treatment. Furthermore, 3D platforms can enable the optimization of treatment parameters, such as the intensity or duration of laser exposure, depending on the type of tissue, location of the tumor, and TME, aiming to guarantee the safety of the healthy tissue around the tumor.

### 4.2 3D tumor models for magnetic hyperthermia nanomedicines screening

**Fig. 5:**
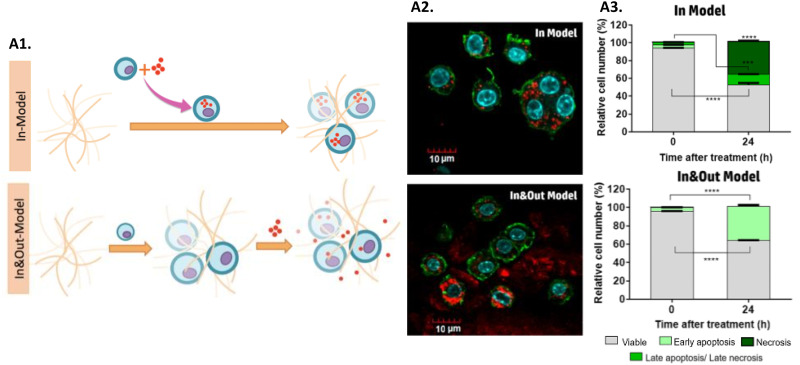
Screening MH nanomedicines in 3D *in vitro* tumor models: (A1) scheme of the two models used in this work: in and In&Out models; (A2) confocal images evaluating the distribution of MNPs in both models, and (A3) cell death mechanisms for both models studied at 0 and 24 hours after the treatment. Reprinted with permission from ref. 278, Copyright 2018 American Chemical Society.

MH allows a high penetration depth and selectivity, which has many benefits when the tumor is localized in sensitive tissues, such as the brain, or more deeply in the body.^87^ Ongoing research and development efforts are focused on optimizing MNPs’ design and targeting strategies. As mentioned before, iron oxide MNPs have been the most explored for MH, which is why they are the most used MNPs for hyperthermia screening in 3D tumor models. To explore the influence that the location, amount, and heterogeneity of iron oxide MNPs have on MH, a study was carried out to evaluate their performance for MH in 3D cell culture gels based on collagen of a murine macrophage RAW-264.7 cell line.^278^ MNPs distribution within the model was manipulated, namely: (i) the In Model had MNPs homogeneously localized only inside the cells, while (ii) the In&Out model had MNPs heterogeneously localized inside and outside the cells (Fig. 5A1 and A2). Results showed that AMF exposure promoted the uptake of MNPs, that could be related to an increase in collagen permeability. Furthermore, it was shown that the cell death mechanisms triggered after treatment were dependent on the iron concentration inside the cells. Additionally, the heterogeneous distribution of particles in the In&Out setup was shown to decelerate the rate of cell death (Fig. 5A3).^278^ These findings are extremely important since it was shown that the distribution and concentration of MNPs in cells influence the treatment, something accessible only using 3D models.

Several studies have also focused on assessing the effectiveness of MNPs for MH, comparing the outcomes of 2D and 3D screening *in vitro* platforms. As an example, SPIONs coated with triarylphosphonium cation (TPP), a mitochondrial-targeting molecule, were tested in liver tumor spheroids with HepG2 cells and 3T3 fibroblasts, and HepG2 monolayer cultures.^279^ Coating SPIONs with TPP allowed them to successfully target the mitochondria, subsequently inhibiting its function and inducing cell death. The uptake of the NPs in the spheroids was lower than in the monolayers. In fact, the authors estimated that a mature spheroid only contained ∼40% of cells in contact with MNPs, which resulted in significant cell death at the proliferative layer of the spheroid. Additionally, 3D spheroids were shown to be more resistant to treatment compared to 2D models, and cancer cells were found to be more sensitive to heat than healthy cells when exposed to the same heating conditions.^279^ Recently, other approach explored MNPs screening in patient-derived organoids.^280^ In this approach, pancreatic ductal adenocarcinoma (PDAC) human-derived organoids were incubated with SPIONs stabilized with a phospholipid-bilayer and used to understand how these nanomedicines penetrated into the 3D model. It was observed that the MNPs were unable to be internalized, being only located in the ECM surrounding the organoid. Furthermore, following MH treatment, organoids viability was significantly lower at 2 hours, when compared to that obtained at 24 hours post-treatment. The authors hypothesized that this behavior could be related to a short-term cytotoxicity caused by the treatment in this type of tumor model. This study also showed how 2D models can be inaccurate in predicting tumor responses and that different cell lines from PDAC had different responses to MH treatment.^280^

Apart from demonstrating the efficiency of MH in eradicating cancer cells within 3D cultures, the presented studies for MH screening in 3D tumor models highlight the potential for improved and targeted outcomes when coupling targeting agents with MNPs. The mechanisms of cell death triggered can vary based on the concentration of iron present in cells,^278^ and can also depend on the properties of the MNPs, frequency, intensity of the AMF, and type of tissue.^118^ Furthermore, the location of the MNPs within the 3D tumor models was shown to influence the efficiency of the treatment. Overall, 3D tumor models showed to be suitable platforms to understand the diffusion mechanisms, penetration, and efficiency of different nanomedicines for MH in different treatment conditions and tissues.

### 4.3 3D tumor models for screening synergistic effects of hyperthermic nanomedicines

**Fig. 6:**
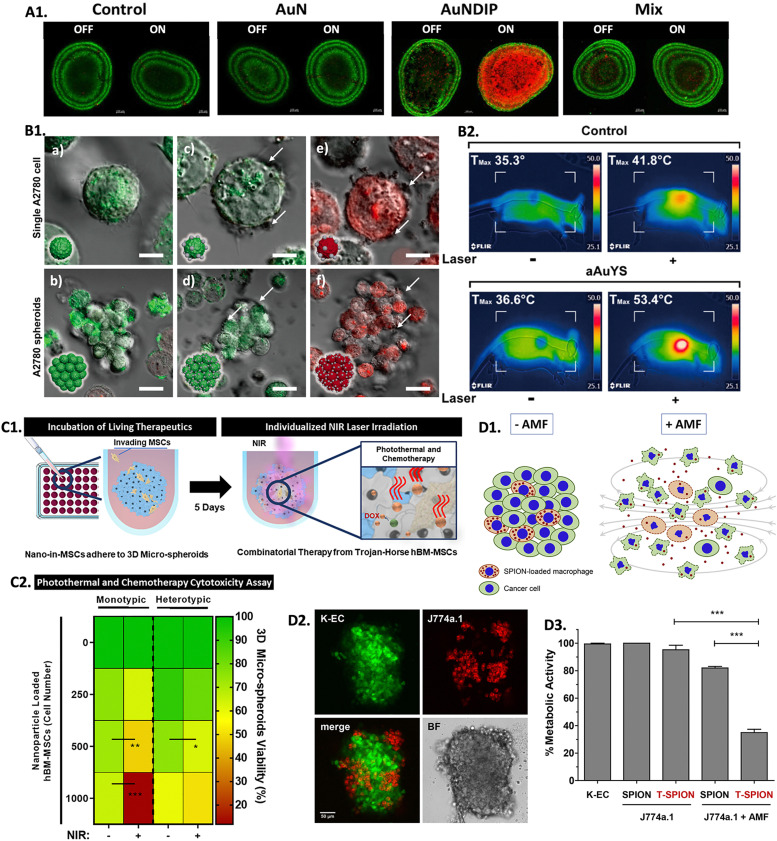
Screening combinational hyperthermia and chemotherapy in 3D *in vitro* tumor models: (A1) live/dead confocal images of control untreated C6 spheroids, and spheroids treated with AuN, AuNDIP, and Mix (DOX-ICG and B-anti G)), with and without laser exposure (scale bar = 100 μm). Reprinted from ref. 287, Copyright (2022), with permission from Elsevier. (B1) Confocal images of A2780 cells and spheroids: (a) and (b) non-treated with aAuYs under laser irradiation; (c) and (d) treated with aAuYS without laser irradiation, and (e) and (f) treated with aAuYS under laser irradiation (scale bars in the upper and lower panels represent 6 and 20 μm, respectively), and (B2) infrared thermal images of a mice treated with DOX and aAuYS submitted to NIR laser, Reprinted with permission from ref. 288. Copyright 2021 American Chemical Society; (C1) scheme representing the performed procedure of hBM-MSCs NPs delivery in 3D spheroids followed by laser irradiation; (C2) heat map enhancing the influence of the number of MSCs loaded with PDA-ICG-DOX NPs in 3D spheroids viability, 3 days after NIR laser treatment (irradiation (NIR +), and without irradiation (NIR −)). Reprinted with permission from ref. 174, Copyright (2021), with permission from Elsevier; (D1) scheme representing the incorporation of macrophage loaded with SPIONs into cancer cells and its effect when submitted to an AMF; (D2) confocal microscopy showing the integration of J774a.1 macrophages (red) in K-EC spheroids (green) (scale bar = 50 μm); and (D3) metabolic activity of re-cultured spheroids containing T-SPION loaded macrophages with or without AMF application after 48 h of treatment. Reprinted with permission from ref. 292, Copyright (2019), with permission from Elsevier.

Hyperthermia exhibits a prominent advantage in its potential to synergistically enhance treatment efficacy when combined with traditional cancer treatments, such as chemotherapy and radiotherapy.^281^ Following thermal exposure,blood flow and vascular permeability increase, leading to improved drug uptake and distribution, and increased cellular stress. Moreover, the interstitial fluid pressure decreases and the mechanisms to repair DNA are inhibited, which prevents irradiated cells from being repaired. Because of this, cancer cells become more susceptible to radiation and chemotherapy after heat treatment.^282–284^ Ongoing research with 3D *in vitro* models is being held to investigate the synergistic effects of hyperthermia when combined with traditional treatments, aiming to find optimal protocols and strategies for integrating heat therapies into multidisciplinary cancer treatment approaches. In combinational PTT and chemotherapy (PTT-CHT), nano-agents can serve as multifunctional platforms. Besides being selective heating sources, they can also function as carriers for chemotherapeutic drugs, enabling controlled and localized drug delivery to the tumor site. The heat generated by the nano-heating agents can enhance drug release from the carriers, improving drug penetration into the tumor and increasing therapeutic efficacy.^285,286^ A study reported the use of gold nanoroses (AuNs) loaded with doxorubicin (DOX), ICG, and a naive chimeric peptide B-anti G (AuNDIPs) for glioma targeting in C6 glioma cells and 3D spheroids.^287^ AuNDIPs were efficiently uptaken both in 2D and 3D models, which was facilitated by the peptide B-anti G. After laser exposure, the authors observed maximum cell death in spheroids treated with AuNDIPs when compared to spheroids treated only with AuNs or a DOX-ICG and peptide Mix solution (Fig. 6A1), which was due to the combinational effect of the DOX, ICG, and the peptide.^287^ In another study, researchers developed gold nanospheres within silica nanocapsules (aAuYSs) to perform PTT-CHT in A2780 ovarian cancer spheroids.^288^ It was shown that only the combination of NIR radiation and aAuYSs significantly reduced the viability of the spheroids (Fig. 6B1). Furthermore, a DOX-resistant cell line (A2780-R) was used to evaluate the synergistic effect of aAuYSs combined with DOX and proved that the number of dead cells was more significant when performing both treatments. This was also confirmed in an *in vivo* xenograft tumor model since only the combination of treatments was capable of inhibiting tumor growth (Fig. 6B2).^288^

Mesenchymal stem cells (MSCs) have been explored as drug vehicles due to their tropism (*i.e.*, the ability to travel to damaged tissues, such as tumors), which has already been demonstrated in several studies.^289,290^ Moreover, it was also demonstrated that hyperthermia can improve the ability of stem cells to penetrate tumors, making them a suitable platform to transport drugs and NPs to tumors to promote localized hyperthermia therapy.^291^ In this scope, a study used monotypic (only one cell type) and heterotypic (multiple co-cultured cell types) 3D breast cancer spheroids to evaluate the potential of human bone-marrow-derived MSCs (hBM-MSCs) as nanocarriers of polydopamine NPs dual-loaded with ICG and DOX (PDA-ICG-DOX) and to evaluate their efficiency for PTT-CHT (Fig. 6C1).^174^ The authors observed that the MSCs were able to adhere to the tumor models efficiently. The combination of PTT and chemotherapy led to a decrease in spheroids viability, which was more pronounced with an increased number of PDA-ICG-DOX NPs loaded MSCs (Fig. 6C2). It was concluded that the heterotypic spheroids were more resistant to treatment than the monotypic spheroids and that the use of MSCs as nanocarriers was more efficient in reducing the viability of tumors than the introduction of free nanomedicines in the 3D tumor models.^174^ These findings are important for understanding how a heterotypic environment can confer more resistance to treatment, mimicking a more *in vivo*-like scenario.

Recently, a study intending to understand the feasibility of macrophages in transporting SPIONs coupled with a toxin (T-SPIONs) to KSHV-infected human endothelial cells (K-EC) spheroids for a combination of MH and chemotherapy (MH-CHT) was carried out (Fig. 6D1 and D2).^292^ Even without being submitted to an AMF, after 48 hours in culture, spheroids containing T-SPIONs presented a significant loss of structure, whereas spheroids with only SPIONs or without SPIONs did not show any significant structural change. The spheroid's structure was even more compromised after treatment with AMF, being more evident when the toxin was coupled to the SPIONs, accompanied by a decrease in the metabolic activity of spheroids (Fig. 6D3).^292^

Hyperthermia before radiotherapy was shown to increase the efficiency of the treatment since heat can selectively target and sensitize cancer cells, making them more vulnerable to radiation-induced DNA damage and cell death. Consequently, hyperthermia can improve tumor response, overcome radioresistance, and potentially reduce the side effectsassociated with high radiation doses.^293^ In an elegant approach, copper sulphide NPs (CuS NPs) were associated at the surface of upconversion NPs (UCNPs) due to manganese dioxide (MnO_2_) coating, forming multifunctional nanoplatforms (UCCM).^294^ Formulated UCCMs were internalized in mouse colon (CT26) spheroids to evaluate internalization in a solid tumor model. It was observed that after 4 hours, CuS NPs were at the center of the spheroid. This allowed MnO_2_ to release Mn^2+^ and oxygen, consequently reducing hypoxia and resistance to radiation. Furthermore, the released Mn^2+^ was shown to be a proper contrast agent for MRI, allowing real-time control of the tumor site that can be helpful for treatment guidance. The therapeutic effect of the UCCM NPs was then evaluated in mice bearing a human liver tumor, which revealed that the tumors shrank compared to non-treated tumors and that the normal tissues were minimally affected. Moreover, the combination of the treatments was far more effective in destroying the cancer cells than the treatments alone.^294^

Studies using 3D *in vitro* models for screening nanomedicines for a combination of hyperthermia and radiotherapy are still a novel approach, thus accounting for the lack of literature reports on this subject. Recent studies continue to rely on standard 2D *in vitro* cultures,^296–299^ which is also important, but there is a need to embrace novel and innovative *in vitro* testing platforms that can more closely recapitulate human tissues response to treatments and ultimately contribute to understanding the underlying mechanisms that contribute to the observed synergistic effects that can accelerate translation to clinical practice. Table 4 showcases studies reporting the application of 3D tumor models as screening platforms to evaluate nano-heating agents for hyperthermia as a stand-alone or combinatory treatment. The examples reported in this section have provided valuable insights into the synergistic interactions between hyperthermia and traditional cancer treatments. This approach allows improved drug penetration, enhanced cancer cell killing, modulation of drug resistance mechanisms, and reduction of the radiation dose and/or chemotherapeutic agents, which is very important to guarantee the safety of healthy tissues. Optimizing treatment parameters, such as exposure time, and nanomedicine or radiation doses, remains crucial. Alongside, recent advances in the combination of hyperthermia with modern and more sophisticated therapies, such as immunotherapy, have been shown to provide better immune recognition and destruction of the tumor since the heat induced by hyperthermia can activate and boost immune cells’ anti- tumoral activity.^125^

**Table 4:** Examples screening the effectiveness of 3D tumor models in predicting the efficiency of nano-heating agents for human and hyperthermia

Heating agent			Heating efficiency^c^	Technique, heating conditions	Tumor model	Dose (μg mL^−1^)	*T* reached during treatment (°C)	Cell death (%) (time after treatment, technique)	Ref.
Core	Coating	Size w/coating (nm)							
DOPA-rGO	Thiol-terminated poly(2-ethyl-2-oxazoline)	100–200, DLS	PCE N.D.	PTT (808 nm, 1.7 W cm^−2^, 5 min)	MCF-7 monolayers and spheroids	75	N.D.	2D model: 97% (24 h, AB)	270
								3D model: 70% (48 h, AB)	
Au nanoshells	Anti-MUC1 aptamer	N.D.	PCE N.D.	PTT, 808 nm, 5 min, single (PTTx1) or double irradiation (PTTx2), 1 h interval	A549 and MCF-7 spheroids in MD	100 μM	N.D.	A549: 31% (PTTx1) and 41% (PTTx2)	272
								MCF-7: 15%^a^ (PTTx1) and 50%^a^ (PTTx2) (48 h, AB)	
rGO	BPEI–PEG	50–60, AFM	PCE N.D.	PTT, 808 nm, 1 W cm^−2^, 10 min	U87MG spheroids in MD	60	N.D.	∼45% (after treatment, live/dead)	274
Black phosohorus nanosheets	HA coating with loaded cypate	187, DLS	PCE ∼ 49% (at 0.5 W cm^−2^)	PTT, 808 nm, 1 W cm^−2^, 3 min	4T1 monolayers and spheroids	25	N.D.	2D model: 60% (24 h, MTT)	276
								3D model: ∼75% (7 days, FC)	
Fe_3_O_4_ NPs	PMAO functiona-lized with glucose	48–67, DLS	SAR = 253 W g_Fe_^−1^ (10^3^ μg_Fe_ mL^−1^, 20 kA m^−1^, 829 kHz)	MH, 13 kA m^−1^, 377.5 kHz, 30 min	ATCC TIB71 culture gels	200	N.D.	In model: ∼40%	278
								In & out model: 38% (24 h, FC)	
								∼96%, both models (48 h, FC)	
Fe_3_O_4_ NPs	Aminosilane	100, N.D.	SAR ∼ 115 W g^−1^ (10^4^ μg_Fe_ mL^−1^)	MH, 300 Gauss, 305 kHz, 10 and 30 min	C6 spheroids in MD	10^4^ b	41–43	10 min: 20%	295
								30 min: 100% (after treatment, live/dead)	
Fe_3_O_4_ NPs	TPP	20, N.D.	SAR = 619 W g^−1^ (340 μg mL^−1^)	MH, 30 A, 300 kHz, 10 min	HepG2 monolayers; 3T3 and HepG2 spheroids	50^b^	N.D.	2D model: ∼50%^a^	279
								3D model: ∼20%^a^ (after treatment, trypan blue)	
SPIONs	Phospholipid bilayer	100, DLS	SAR = 406 W g_Fe_^−1^	MH, 40–47 kA m^−1^	PANC-1 monolayers and PDAC organoids	225^b^	N.D.	2D model: 27%	280
				270 kHz, 30 min				3D model: 48% and 13% (2 h, 24 h, CellTiter-Glo®)	
Gold nanoroses (AuNs) loaded with DOX and ICG	Peptide B-anti G	295, (just AuNs) DLS	N.D.	PTT-CHT, 808 nm, 2 W cm^−2^, 10 min	C6 monolayers and spheroids	100	N.D.	2D model: ∼85%	287
								3D model: ∼88% (24 h, MTT)	
PDA loaded with DOX and ICG NPs	hBM-MSCs	328, DLS	PCE ∼ 90%	PTT-CHT, 808 nm, 1.6 W cm^−2^, 5 min	Monotypic (MDA-MB-231) and heterotypic (MDA-MB-231 and BCAFs) spheroids	500	N.D.	Monotypic spheroids: ∼60%^a^	174
								Heterotypic spheroids: ∼40%^a^ (3 days, CellTiter-Glo®)	
SPIONs coupled with a toxin	Silica coating; loaded in mouse macro-phages (J774a.1 cells)	N.D.	N.D.	MH-CHT, 4.8 kA m^−1^, 779 kHz, 40 min	K-EC spheroids	30 μg/10^5^ cells	N.D.	∼65%^a^ (48 h, WST-1)	292

a Value extracted from graphs.

b Fe concentration.

c The conditions that are not indicated are the same as the heating conditions; N.D. – non described; MD – microfluidic device; DOPA: dopamine; rGO: reduced graphene oxide; BPEI: branched polyethyleneimine; AFM: atomic force microscopy; HA: hyaluronic acid; PMAO: poly(maleic anhydride-*alt*-1-octadecene; TPP: triphenylphosphonium cation; PDAC: pancreatic ductal adenocarcinoma; PDA: polydopamine; hBM-MSCs: human bone-marrow derived mesenchymal stem cells; BCAFs: breast cancer-associated fibroblasts.

Besides being used to predict the efficiency of the treatment, 3D *in vitro* models can be useful platforms to assist the design process of the nano-agents for parameters such as size, morphology, coating, stabilization, and targeting optimization, in a specific and relevant tumor context. A study using colorectal cancer spheroids was carried out to understand how the internalization of NPs was affected by their size, surface, and bulk characteristics.^300^ The authors considered poly(styrene) NPs with different sizes and different coatings and concluded that unmodified NPs with smaller sizes had more efficiency in accumulating at the core of the spheroids. Regarding surface charge, it was shown that better penetration ability was achieved with positively charged NPs, due to the presence of negatively charged elements in the ECM that can repel negative charges. Moreover, it was discussed that lower molecular weight NPs have greater efficiency in penetrating tumors.^300^ In another study, the influence of the shape of the NPs on penetrating 3D HeLa tumor spheroids was assessed.^301^ Sphere, long rod, and short rod-shaped NPs were studied, and the uptake efficiency in spheroids was shown to be strongly dependent on the length-to-width ratio. The authors concluded that NPs with higher length-to-width ratios had more difficulty penetrating tumors than those with lower length-to-width ratios. Additionally, the uptake efficiency was different when considering 3D tumor spheroids and monolayers.^301^ The presented studies emphasize the importance of considering the physicochemical properties of NPs during the design phase, aiming to optimize their hyperthermic efficiency.

## 5. Conclusions and future challenges

With the developments achieved in the last few years, organotypic 3D models have emerged as feasible biomimetic platforms, capable of replacing the standard 2D models that continue to be widely explored for screening hyperthermic nanomedicines. These advanced biomimetic models enable a closer examination of the effects induced by heat within tumors, taking into consideration factors such as tri-dimensionality, size, composition of the TME, availability of nutrients and oxygen, intercellular signaling, and nanomedicines properties. The tissue-specific characteristics of 3D tumor models that allow the incorporation of several ECM components and other cell types can help in the design of cell-specific cancer platforms to optimize treatment outcomes. Due to the inherent unpredictability, complexity, and specificity of tumors for each patient, this approach allows for personalized treatment models that offer improved accessibility and predictive capabilities in accessing the response to hyperthermia therapies. Despite these improvements at the preclinical stage, the protocols of hyperthermic procedures must be standardized, as well as the characterization techniques.^302,303^ As discussed above (Tables 1, 2 and 4), highly relevant information regarding treatment procedures is lacking from literature reports, which may impede the establishment of standardized protocols.

It is pertinent to highlight the importance of considering the design of more complex cellular 3D *in vitro* structures (*i.e*., regarding ECM components, cellular types, incorporation of vascularization and diffusion mechanisms, *etc.*) in the future, aiming to provide more realistic screening platforms. As it was understood by the reported studies, spheroids are the predominant model as they are the most simple and cost-effective testing platforms. Certainly, organoids have gained increasing attention recently, offering more complexity and a more *in vivo*-predictive response to treatments. This development aims to facilitate the translation of hyperthermic nanomedicines into clinical practice. Furthermore, combining spheroids or organoids with tumor-on-a-chip platforms and 3D bioprinting techniques can increase the complexity of the models, as these allow to introduce components of the tumor vasculature and flow dynamics/mechanisms. Also, some studies reported here provided both 3D *in vitro* and *in vivo* assessment of the hyperthermic nanomedicines, which enables to confirm that these models can provide closer *in vivo* responses and consequently contribute to accelerating hyperthermic nanotherapies preclinical validation and subsequent transition to clinical practice.

Even though hyperthermia demonstrates notable efficacy in tumors, its widespread clinical implementation is being delayed by prominent limitations, including inadequate penetration and heterogeneous distribution of heating agents in tumors, and insufficient control over temperature regulation at the tumor site. As shown and discussed herein, researchers have been focused on studying different carriers and targeting agents capable of improving the internalization of heating agents in the tumor, which have been shown to provide a more localized treatment with better therapeutic outcomes. Approaches that involve focalized delivery are also envisioned to be further explored in the future.

Regarding temperature control, there is a need to have a reliable method for real-time assessment, thereby mitigating damage to healthy tissues around the tumor mass. However, invasive devices (*e.g.*, optical fibers and thermocouples) currently represent the prevailing method for temperature control in hyperthermia procedures.^304^ As noted in the examples documented in Table 4, a substantial number of studies did not present the temperature that the 3D models reached during the treatment, which can be due to the difficulty in controlling the temperature in real-time. Currently, non-invasive thermometry systems based on MRI,^305,306^ magnetic particle imaging (MPI),^307^ or fluorescence thermometry^308,309^ are being developed, representing an outstanding breakthrough in the field by allowing temperature control in real-time and in a minimally invasive way. However, 3D tumor models are challenging tools when it comes to obtaining temperature maps with adequate spatial resolution and for imaging. With increasing tumor size, light has less penetration ability, as it gets more scattered. Consequently, the ability to acquire temperature maps or high-resolution images declines, particularly considering deeper regions within the tumor. Furthermore, larger tumor sizes can be a barrier to prompt acquisition of images, thus compromising the real-time control of the heat therapy.^23,304,310^ To address these limitations, researchers have been exploring strategies to confer nanomedicines with theranostic capabilities, *i.e.*, the ability to perform an image-guided therapy.^311–313^ By using nanomedicines for temperature control, the problem of spatial resolution can be addressed, but imaging techniques capable of capturing such fine-scale response are needed.^310^ MRI has a spatial resolution in the order of the millimeters,^314^ which can be crucial to provide high-resolution temperature maps, as the ones obtained in.^315,316^ By tuning the transition temperature of MNPs to room temperature, it is possible to use those nanomaterials as temperature sensors in the range of hyperthermia.^317^ Consequently, these agents can be simultaneously used as contrast agents and thermometers for MRI during hyperthermia treatments. However, MRI-based thermometry still faces challenges with low signal-to-noise ratio and inaccurate temperature calibrations.^318^

**Fig. 7:**
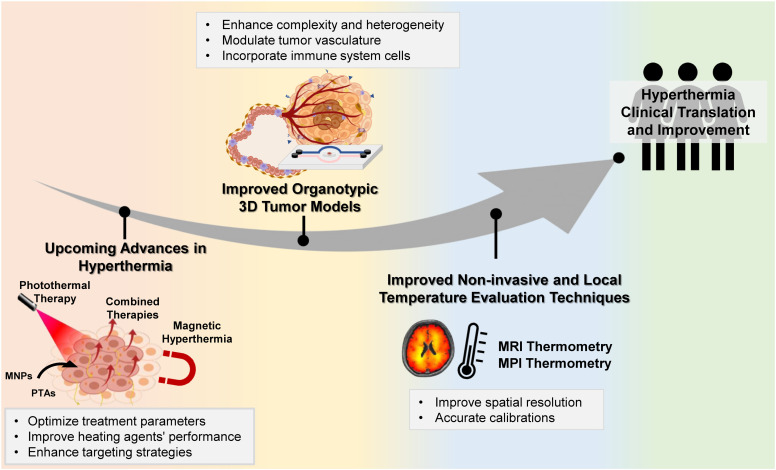
Developments in hyperthermia for clinical translation: progressive advancements of hyperthermia treatments, 3D tumor models, and non-invasive thermometry techniques that can accelerate the translation of hyperthermia to clinical practice.

Integrating advancements in 3D *in vitro* tumor models with emerging hyperthermia techniques and non-invasive 3D thermometry systems could mitigate the disparities in readouts between preclinical and clinical trials for screening advanced anti-cancer therapeutics (Fig. 7). This convergence of technologies holds promise for enhancing the translational relevance of treatments, foreseeably facilitating their widespread use in clinical practice.

## Conflicts of interest

The authors declare no competing interests.

## Supplementary Material
